# Childhood adiposity and risk of type 1 diabetes: A Mendelian randomization study

**DOI:** 10.1371/journal.pmed.1002362

**Published:** 2017-08-01

**Authors:** J. C. Censin, Christoph Nowak, Nicholas Cooper, Peter Bergsten, John A. Todd, Tove Fall

**Affiliations:** 1 Department of Medical Sciences, Molecular Epidemiology and Science for Life Laboratory, Uppsala University, Uppsala, Sweden; 2 Juvenile Diabetes Research Foundation/Wellcome Trust Diabetes and Inflammation Laboratory, Department of Medical Genetics, Cambridge Institute for Medical Research, National Institute for Health Research Cambridge Biomedical Research Centre, University of Cambridge, Cambridge, United Kingdom; 3 Department of Medical Cell Biology, Uppsala University, Uppsala, Sweden; 4 JDRF/Wellcome Trust Diabetes and Inflammation Laboratory, Wellcome Trust Centre for Human Genetics, Nuffield Department of Medicine, NIHR Oxford Biomedical Research Centre, University of Oxford, Oxford, United Kingdom; University of Cambridge, UNITED KINGDOM

## Abstract

**Background:**

The incidence of type 1 diabetes (T1D) is increasing globally. One hypothesis is that increasing childhood obesity rates may explain part of this increase, but, as T1D is rare, intervention studies are challenging to perform. The aim of this study was to assess this hypothesis with a Mendelian randomization approach that uses genetic variants as instrumental variables to test for causal associations.

**Methods and findings:**

We created a genetic instrument of 23 single nucleotide polymorphisms (SNPs) associated with childhood adiposity in children aged 2–10 years. Summary-level association results for these 23 SNPs with childhood-onset (<17 years) T1D were extracted from a meta-analysis of genome-wide association study with 5,913 T1D cases and 8,828 reference samples. Using inverse-variance weighted Mendelian randomization analysis, we found support for an effect of childhood adiposity on T1D risk (odds ratio 1.32, 95% CI 1.06–1.64 per standard deviation score in body mass index [SDS-BMI]). A sensitivity analysis provided evidence of horizontal pleiotropy bias (*p* = 0.04) diluting the estimates towards the null. We therefore applied Egger regression and multivariable Mendelian randomization methods to control for this type of bias and found evidence in support of a role of childhood adiposity in T1D (odds ratio in Egger regression, 2.76, 95% CI 1.40–5.44). Limitations of our study include that underlying genes and their mechanisms for most of the genetic variants included in the score are not known. Mendelian randomization requires large sample sizes, and power was limited to provide precise estimates. This research has been conducted using data from the Early Growth Genetics (EGG) Consortium, the Genetic Investigation of Anthropometric Traits (GIANT) Consortium, the Tobacco and Genetics (TAG) Consortium, and the Social Science Genetic Association Consortium (SSGAC), as well as meta-analysis results from a T1D genome-wide association study.

**Conclusions:**

This study provides genetic support for a link between childhood adiposity and T1D risk. Together with evidence from observational studies, our findings further emphasize the importance of measures to reduce the global epidemic of childhood obesity and encourage mechanistic studies.

## Abstract 2

Tove Fall and colleagues, using a Mendelian randomization study, show support for the link between childhood adiposity and increased risk of type 1 diabetes.

## Author summary

**Why was this study done?:**

- Type 1 diabetes (T1D) rates have been increasing globally in recent decades; however, the contributing factors to this increase have not been established.
- Childhood obesity has been suggested to contribute to T1D risk. Since obesity rates in children have been increasing in recent years, they may explain part of the rise in T1D rates. However, conventional observational epidemiologic studies are limited in explaining causality because of possible bias from unmeasured confounding and reverse causation.

**What did the researchers do and find?:**

- We investigated if genetic variants associated with childhood body mass index (BMI) were also associated with T1D risk in 5,913 T1D and 8,828 control samples.
- We studied the effect of childhood adiposity on T1D using a Mendelian randomization analysis framework, which uses genetic variants associated with an exposure as instruments to test for effects on an outcome. Since alleles are randomly allocated before birth, Mendelian randomization studies limit bias from confounding factors and reverse causation. We used sensitivity analyses to control for violations of Mendelian randomization assumptions.
- We estimated that a genetically predicted increase in childhood BMI by 1 standard deviation is associated with an average 32% increased risk of T1D (odds ratio 1.32, 95% confidence interval 1.06–1.64).

**What do these findings mean?:**

- This study provides genetic support for a link between childhood adiposity and T1D risk, which could help explain part of the increase in T1D rates.
- However, as the underlying genes and adiposity-increasing mechanisms for most of the genetic variants included in the study are not known, there is a possibility that the genetic variants affect T1D risk through other mechanisms than through adiposity, which would limit causal inference.
- Together with previous studies, our findings reinforce the need to address rising childhood overweight and obesity rates, whose adverse consequences in adulthood pose serious challenges to personal and population health.

## Introduction

The incidence of type 1 diabetes (T1D) in children is rising globally, with reports of annual increases over the last decades of up to 3%–4% in Europe [1,2] and 5% in North America [1]. The reasons for this rapid increase in T1D remain obscure [3]. Years before overt T1D develops, autoantibodies against pancreatic islet cells can be detected [4], and the presence of at least 2 types of autoantibodies is diagnostic of early T1D [5]. The etiology of T1D is generally thought to be autoimmune, where a chronic T lymphocyte-mediated autoimmune reaction is presumed to destroy the insulin-producing beta cells in the pancreas following a trigger event such as an infection [5–7]. This concept, however, has been challenged by recent observations [8,9]. The heritability of T1D is estimated to be 69% [10], but genetic variation alone cannot explain the recent rapid increase. Several environmental risk factors and potential triggers of the autoimmunity have been investigated, including intake of cow's milk, breastfeeding practices, socioeconomic status, and enteroviral infections, but causal effects have not been proven [3].

In 1975, Baum et al. [11] suggested that increased weight gain in infancy is linked to the development of T1D. Later theories, including the “accelerator hypothesis” [12] and the “overload hypothesis” [13] suggest that increased insulin resistance and insulin demand, as seen in, for example, obese individuals, cause beta cell stress and apoptosis and thereby induce autoimmunity. Both theories argue that this would cause T1D to present at a younger age [12,13]. In support of this hypothesis, the insulin sensitivity-increasing genetic variant Pro12Ala in *PPARG* was found to be associated with a lower risk of T1D [14]. For the past decades, the prevalence of childhood obesity has increased by approximately 0.5% annually in the United States and 1% per year in England, Scotland, and Wales [15]. Currently, an estimated 6.6% of all children under the age of 5 years are obese worldwide [16]. Given increasing global rates of childhood obesity, an effect of adiposity on the rising incidence of T1D has been suggested [15,17]. In some countries, like the United Kingdom, the increase in childhood obesity has started to plateau [18]—as has the incidence of T1D [2]. However, several observational studies have failed to find a link between childhood adiposity and T1D risk [19] or age of onset [20–22]. One problem with studying the association of adiposity and T1D is that weight loss is a common symptom in T1D, and prospective studies are therefore needed. Another problem is that T1D is an infrequent disease, whilst large sample sizes are needed for robust inference in prospective designs. Verbeeten et al. [23] performed a meta-analysis of studies measuring body mass index (BMI) before T1D diagnosis. The study found an increased risk of T1D in obese children estimated to an odds ratio (OR) of 2.03 (95% confidence interval [CI] 1.46–2.80) and evidence of a dose-response relationship with an OR of 1.25 (95% CI 1.04–1.51) per unit increase in age- and sex-specific standard deviation score of childhood body mass index (SDS-BMI). Another meta-analysis showed positive correlations between higher birth weight as well as rapid weight gain in infants and T1D risk [24]. Further, a recent study in 1,117 autoantibody-positive children found that elevated BMI over time was associated with higher risk of progression to T1D in youth [25].

Common issues with observational studies such as confounding and reverse causation can be avoided by using instrumental variable (IV) analysis, in which an IV is used as a nonconfounded proxy for the exposure of interest (here adiposity) [26]. In Mendelian randomization (MR) analysis, genetic variants are used as IVs. Since allelic variants are randomly allocated at conception, their lifelong effects precede the outcome (T1D) and minimize bias from reverse causation and confounding. This "quasirandomization" before birth has been reported able to predict the results of clinical trials [27,28]. However, the robustness of MR relies heavily on the validity of the genetic variants used as IVs. One assumption is the absence pleiotropic effects: the IV should not affect the outcome through factors other than the exposure. This assumption cannot be tested completely, but recently developed sensitivity analyses can indicate and correct for violations [29].

Our aim was to use the largest available samples with genetic association results to test the hypothesis of a role for childhood adiposity in childhood T1D etiology. We applied a comprehensive 2-sample MR framework to investigate the effect of childhood adiposity on T1D diagnosis before age 17 and found evidence in line with a role in T1D development.

## Methods

### Ethical approval

Cohorts participating in the genome-wide association studies (GWASs) used in the present study received ethics approval from local institutional review boards and informed written consent from all participants.

### Genetic instrument

To construct a genetic IV for childhood adiposity, we used data from the largest meta-analysis of GWASs of childhood adiposity to date (by Felix et al. [30]), in which adiposity was measured as SDS-BMI at the oldest age of available measurement in children of European descent between 2–10 years of age. The design included up to 35,668 children from 20 studies in the discovery phase and up to 11,873 children from 13 studies in the replication phase. The study identified 15 loci associated with childhood BMI that explained 2.0% of the observed variance [30]. We used the following criteria to select variants as IVs: (1) genome-wide association (*p* < 5 × 10^−8^) with childhood SDS-BMI (15 single nucleotide polymorphisms [SNPs]); (2) suggestive association with childhood adiposity (*p* < 5 x 10^−6^ in both discovery and joint analysis) and association with adult BMI (Bonferroni-adjusted *p* < 0.0055) [31] with consistent effect direction in children and adults (7 SNPs); and (3) genome-wide significant association with adult BMI [31] and childhood BMI (Bonferroni-adjusted *p* < 5.2 x 10^−4^) with consistent effect direction (22 SNPs). There were substantial overlaps of the latter with the 2 former categories, and we kept 1 marker per genetic loci, with preference to the 2 former categories resulting in a total of 27 SNPs. However, the variant rs3888190 was not available in the T1D dataset described below and hence excluded, resulting in 26 SNPs. We conducted 2 analyses, using the combined IVs described above (23 SNP score after further exclusions, see below) and a restricted instrument of variants identified in category 1 (13 SNP score).

To reduce the risk of violating the IV assumptions, we investigated the IVs' associations with 3 biologically plausible confounders—birth weight, smoking, and years of education. Summary-level effect estimates and standard errors (SEs) for birth weight were obtained from the EGG (Early Growth Genetics) Consortium [32], for “ever smoker” from the Tobacco and Genetics Consortium [33], and for years of education from the Social Science Genetic Association Consortium [34]. All effects were aligned to the BMI-increasing allele.

Three SNPs were associated with a potential confounder at a Bonferroni-corrected *p*-value (*p* < 0.05 / 26; for 26 SNPs) and were excluded from the analysis (rs13387838, smoking; rs1808579, education; and rs13253111, birth weight), resulting in a final analytical set of 23 SNPs and 13 SNPs respectively.

### Association with T1D

Summary-level association results for the final set of 23 SNPs were extracted from the largest publicly available T1D GWAS, which was conducted by Cooper et al. [35]. Cooper et al. reanalyzed the data from Barrett et al. [36] with imputation to the 1000 Genomes Project phase III panel in 5,913 samples from individuals with T1D and 8,828 reference samples from individuals of European ancestry. In brief, samples from individuals with T1D were derived from the Wellcome Trust Case Control Consortium, the 1958 Birth Cohort, and from the UK Genetic Resource for Investigating Diabetes (GRID) collection of the Juvenile Diabetes Research Foundation/Wellcome Trust Diabetes and Inflammation Laboratory project [37]. The average age at diagnosis of T1D was 7.8 years (SD 3.9) in the Wellcome Trust Case Control Consortium and 7.2 years (SD 3.8) in GRID. T1D criteria were diagnosis before age 17 and continuous insulin treatment since diagnosis for more than 6 months. Individuals with monogenic types of diabetes were excluded [37,38]. A population-based sample from the UK Blood Services, the 1958 Birth Cohort, and the Wellcome Trust Case Control Consortium was used as the control cohort and combined with a study of bipolar disease to maximize power (as implemented in the original analysis [36]). Table 1 summarizes the final dataset, with details on genotyping platform and baseline characteristics. The quality metrics “info score” from the imputation software IMPUTE2 was >0.6 for all variants in the T1D data. For 1 SNP (rs3829849) unavailable in the T1D data, we used the proxy (rs62578127) with an r^2^ of 1 based on SNiPA (http://snipa.helmholtz-muenchen.de/snipa3/). Data for the T1D GWAS are deposited in the Dryad repository: http://dx.doi.org/10.5061/dryad.ns8q3 [39].

**Table 1:** Description of sample sources for the T1D GWAS.

Status	*N*	Source	Genotyping	Inclusion criteria	Exclusion criteria	Age	% Female	Reference
T1D	3,983	UK GRID	Illumina HumanHap550v3 (550k) Infinium Beadchip	Age of diagnosis 6 months to 16 years, insulin dependent for >6 months, resident in mainland UK, and self-identified white European	Participated in WTCCC GWAS	7.8 years (SD = 3.9) at diagnosis	47	[36]
T1D	1,930	WTCCC	Affymetrix 500K	Age of diagnosis <17 years, insulin dependent since diagnosis for >6 months, and self-identified white European		7.2 years (SD = 3.8) at diagnosis	49	[40]
Controls	3,999	1958BC	Illumina HumanHap550v3 (550k) Infinium Beadchip	Self-reported white ethnicity and representative of gender and geographical region		100% 40–49 years	51	[36]
Controls	1,455	UKBS	Affymetrix 500K	Resident in England, Scotland, or Wales and self-identified white European		37% <40 years; 27% 40–49 years; 28% 50–59 years; 8% >60 years	52	[39]
Controls	1,490	1958BC	Affymetrix 500K	Self-reported white ethnicity and representative of gender and geographical region		100% 40–49 years	48	[40]
Controls	1,884	Bipolar	Affymetrix 500K	Individuals with bipolar disease, age > 16 years, resident in mainland UK, and of European descent		30% <40 years; 29% 40–49 years; 24% 50–59 years; 17% >60 years	62	[40]

1958BC, 1958 Birth Cohort; GRID, Genetic Resource for Investigating Diabetes; GWAS, genome-wide association study; SD, standard deviation; T1D, type 1 diabetes; UKBS, UK Blood Services; WTCCC, Wellcome Trust Case Control Consortium.

### Main analysis

For all SNPs, we used the effect estimate and SE estimated in the childhood BMI GWAS (Table 2). For all outcomes and sensitivity analyses, effects were aligned to the BMI-increasing allele reported in [41]. Inverse-variance weighted (IVW) estimates for the effect of adiposity on T1D risk were calculated using the analysis code provided in [26].

**Table 2:** Association of adiposity-related genetic variants with childhood body mass index and type 1 diabetes.

SNP				SDS-BMI^1^		T1D^2^	
Marker name	Nearest gene	EA	EAF	Beta (95% CI)	*p*-Value	OR (95% CI)	*p*-Value
rs13130484^3^	*GNPDA2*	T	0.42	0.067 (0.053–0.081)	1.58 x 10^−23^	1.064 (1.013–1.117)	0.01
rs11676272	*ADCY3*	G	0.46	0.068 (0.054–0.082)	7.12 x 10^−23^	1.060 (1.009–1.113)	0.02
rs4854349	*TMEM18*	C	0.83	0.090 (0.072–0.108)	5.41 x 10^−22^	1.055 (0.989–1.126)	0.10
rs543874	*SEC16B*	G	0.19	0.077 (0.059–0.095)	2.20 x 10^−19^	1.031 (0.973–1.094)	0.30
rs7132908	*FAIM2*	A	0.36	0.066 (0.050–0.082)	1.57 x 10^−18^	1.010 (0.960–1.063)	0.70
rs1421085	*FTO*	C	0.43	0.059 (0.045–0.073)	4.53 x 10^−16^	0.991 (0.944–1.041)	0.72
rs12429545	*OLFM4*	A	0.11	0.076 (0.056–0.096)	2.08 x 10^−14^	1.045 (0.968–1.128)	0.26
rs987237	*TFAP2B*	G	0.18	0.062 (0.044–0.080)	1.80 x 10^−12^	0.987 (0.927–1.051)	0.68
rs12041852	*TNNI3K*	G	0.45	0.046 (0.032–0.060)	2.28 x 10^−10^	1.057 (1.008–1.107)	0.02
rs6567160	*MC4R*	C	0.24	0.050 (0.034–0.066)	1.21 x 10^−9^	1.039 (0.981–1.099)	0.19
rs8092503	*RAB27B*	G	0.22	0.045 (0.029–0.061)	8.17 x 10^−9^	0.968 (0.914–1.024)	0.26
rs3829849 (rs62578127)^4^	*LMX1B*	T	0.37	0.041 (0.027–0.055)	8.81 x 10^−9^	1.008 (0.960–1.059)	0.75
rs7550711	*GPR61*	T	0.03	0.105 (0.068–0.142)	4.52 x 10^−8^	0.996 (0.845–1.174)	0.96
rs17309930	*BDNF*	A	0.18	0.045 (0.027–0.063)	1.41 x 10^−7^	0.974 (0.917–1.035)	0.40
rs2590942	*NEGR1*	T	0.81	0.047 (0.029–0.065)	1.91 x 10^−7^	1.040 (0.978–1.105)	0.21
rs13107325^3^	*SLC39A8*	T	0.08	0.081 (0.050–0.112)	3.79 x 10^−7^	0.981 (0.890–1.082)	0.70
rs10151686	*PRKD1*	A	0.05	0.096 (0.059–0.133)	6.99 x 10^−7^	1.006 (0.886–1.143)	0.93
rs11079830	*HOXB6*	A	0.60	0.034 (0.020–0.048)	1.98 x 10^−6^	1.013 (0.965–1.064)	0.60
rs4569924	*GALNT10*	T	0.44	0.032 (0.018–0.046)	3.48 x 10^−6^	1.011 (0.963–1.062)	0.66
rs8046312	*GPR139*	A	0.83	0.042 (0.024–0.060)	3.97 x 10^−6^	0.964 (0.903–1.028)	0.26
rs1441264	*MIR548A2*	A	0.63	0.032 (0.017–0.048)	4.46 x 10^−5^	0.991 (0.942–1.043)	0.73
rs29941	*KCTD15*	G	0.67	0.030 (0.014–0.045)	2.42 x 10^−4^	0.987 (0.938–1.039)	0.62
rs3810291	*ZC3H4*	A	0.66	0.032 (0.015–0.049)	2.85 x 10^−4^	0.918 (0.870–0.967)	0.001

CI, confidence interval; EA, effect allele; EAF, effect allele frequency (from 1000 Genomes Project, http://www.internationalgenome.org/); OR, odds ratio; SDS-BMI, age- and sex-specific standard deviation score of childhood body mass index; SNP, single nucleotide polymorphism; T1D, type 1 diabetes.

^1^Felix et al. [30], sex- and age-adjusted standard deviation scores of body mass index

^2^T1D OR and CI from Cooper et al. [35]

^3^Excluded in sensitivity analysis, has *p* < 0.01 with years of education

^4^Proxy was used in T1D data

### Sensitivity analysis

There is no one "gold standard" way of conducting an MR study. All available methods have advantages and shortcomings that balance power, precision, and adjustment for bias. We therefore carried out several sensitivity methods in addition to IVW MR to provide a comprehensive causal inference framework of the available evidence in an epidemiologic context. We compared results from the main analysis with those obtained in reanalysis with more robust methods, which are less powerful but rely on fewer assumptions than IVW MR. The following calculations and graphs were implemented using the software code provided in [26,29].

#### Assessment of the IV assumptions

We used visual assessment of heterogeneity and a formal heterogeneity test to assess the compatibility of IV estimates based on individual genetic variants. Heterogeneity among such estimates indicates that analysis based on different SNPs yields different estimates, which is indicative of pleiotropic effects. We further used funnel plots of IV precisions (1/SE_IV_) against the IV estimates, which should form a symmetrical funnel shape (i.e., more precise estimates are less variable). Asymmetries in this type of plot indicate directional pleiotropy. We formally tested this with the intercept test in MR Egger regression and further assessed the association of the 2 SNP sets with birth weight, education, and smoking in IVW MR.

#### Robust analysis methods

We first reanalyzed the data with a likelihood-based method [42], which in case of considerable imprecision in the estimates of the association of the genetic variants with the exposure gives appropriately sized CIs. We then obtained IVW estimates using random effects models, which provide a more appropriate model in the presence of heterogeneity. Third, we used MR Egger regression, allowing for asymptotically consistent estimates even if all the genetic instruments are invalid based on the assumption of the Instrument Strength Independent of Direct Effect (InSIDE) postulate (i.e., the potential pleiotropic effects are not related to instrument strength [43]). In addition, the simple and weighted median-based methods were applied. Median methods provide accurate estimates if 50% or more of the included variants are valid instruments. We also calculated the effect of childhood adiposity on birth weight, years of education, and smoking using similar MR methods as in the main analysis. For the statistical analyses, R version 3.2.3 [44] was used as well as packages “shape” [45], “meta” [46], “Hmisc” [47], and “MendelianRandomization.” For plots, packages “ggplot2,” “gridExtra,” and “grid” were used. We also reran the analysis excluding 2 SNPs (rs13130484 and rs13107325) associated with lower education at a nominal level (*p* < 0.01), as proposed in [26].

#### Multivariable Mendelian randomization

We further applied regression models of the association of adiposity SNPs with T1D correcting for the genetic association with potential confounders birth weight, smoking, and education, using the methods described in [48], in which the regression model is weighted by the inverse SE of the T1D estimate and the intercept is null.

## Results

### Main analysis

The IVW method provided evidence for a positive association between SDS-BMI and T1D, with an OR of 1.32 (95% CI 1.06–1.64; that is an average 32% increased risk of T1D per SD increase in BMI) for the larger SNP set (23 SNPs) and an OR of 1.55 (95% CI 1.21–1.99) for the smaller set of 13 SNPs.

### Sensitivity analysis

#### Assessment of the IV assumptions

**Fig 1:**
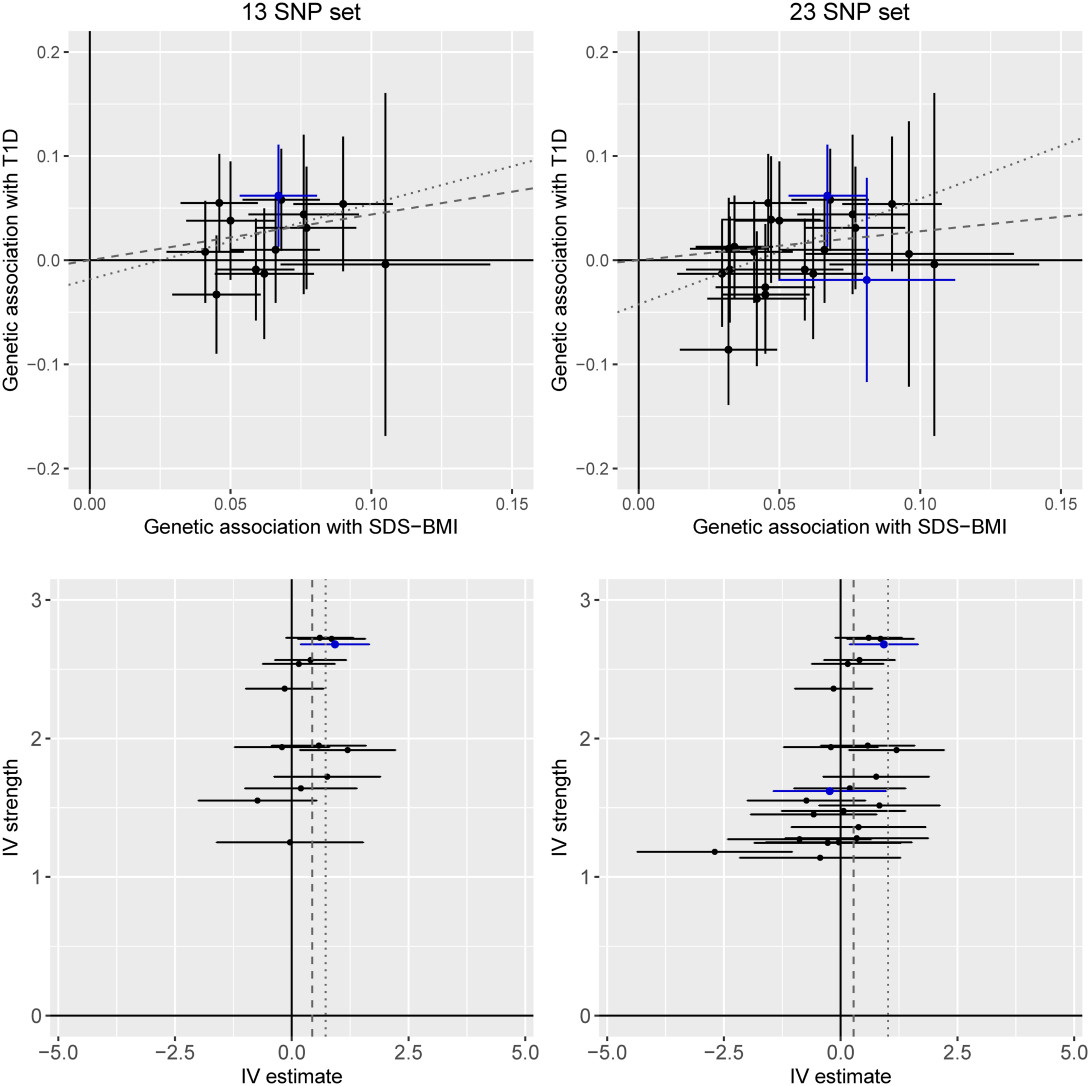
Sensitivity analysis. Upper panels show scatter plots of genetic association with type 1 diabetes over genetic associations with SDS-BMI. Lines represent 95% confidence intervals. The lower panels show funnel plots of instrumental variable precision against instrumental variable estimates for genetic associations between SDS-BMI and type 1 diabetes. In all panels, the dashed line represents slopes/estimates for inverse-variance-weighted analysis, and the dotted line represents the slope from MR Egger regression. rs13130484 and rs13107325 were nominally associated (*p* < 0.01) with education and marked with blue color. IV, instrumental variable; SDS-BMI, age- and sex-specific standard deviation score of childhood body mass index; SNP, single nucleotide polymorphism; T1D, type 1 diabetes.

**Fig 2:**
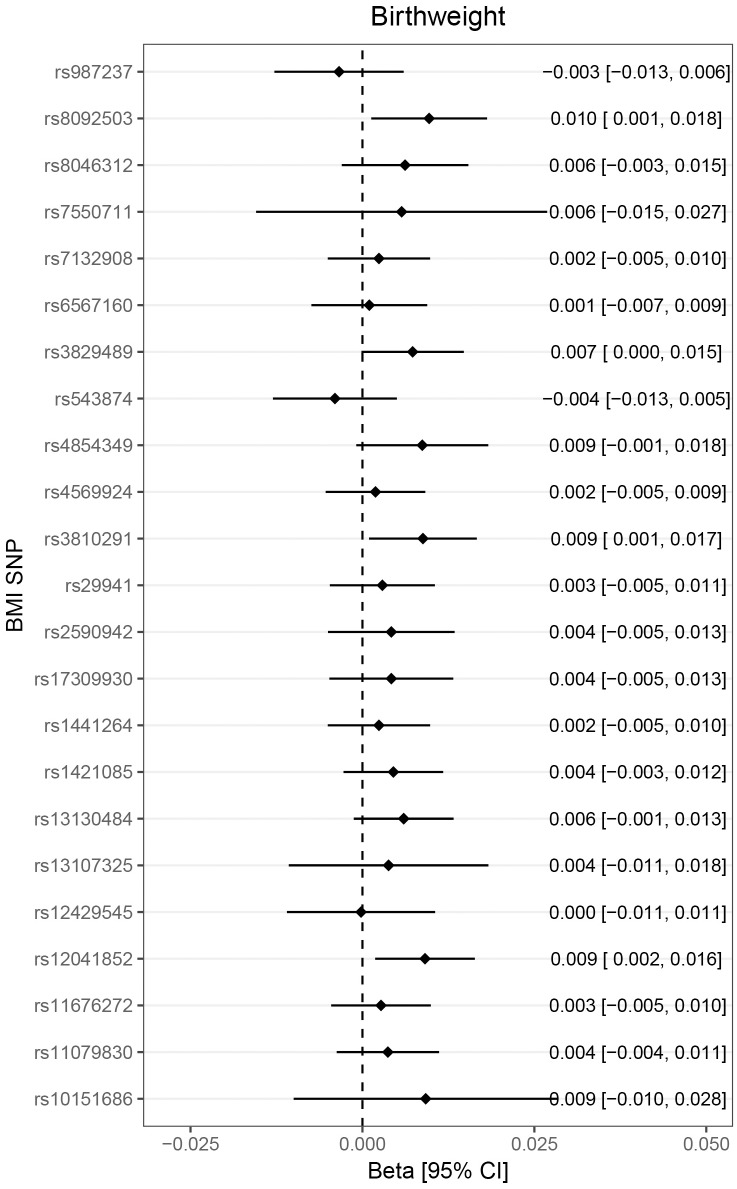
Association of childhood adiposity-related genetic variants with birth weight. Lines represent 95% confidence intervals. Note that because of the large number of SNPs investigated, the threshold for nominal significance was set to *p* < 0.01. No SNP was alone associated with birthweight, but the combined score showed a positive association. BMI, body mass index; CI, confidence interval; SNP, single nucleotide polymorphism.

**Fig 3:**
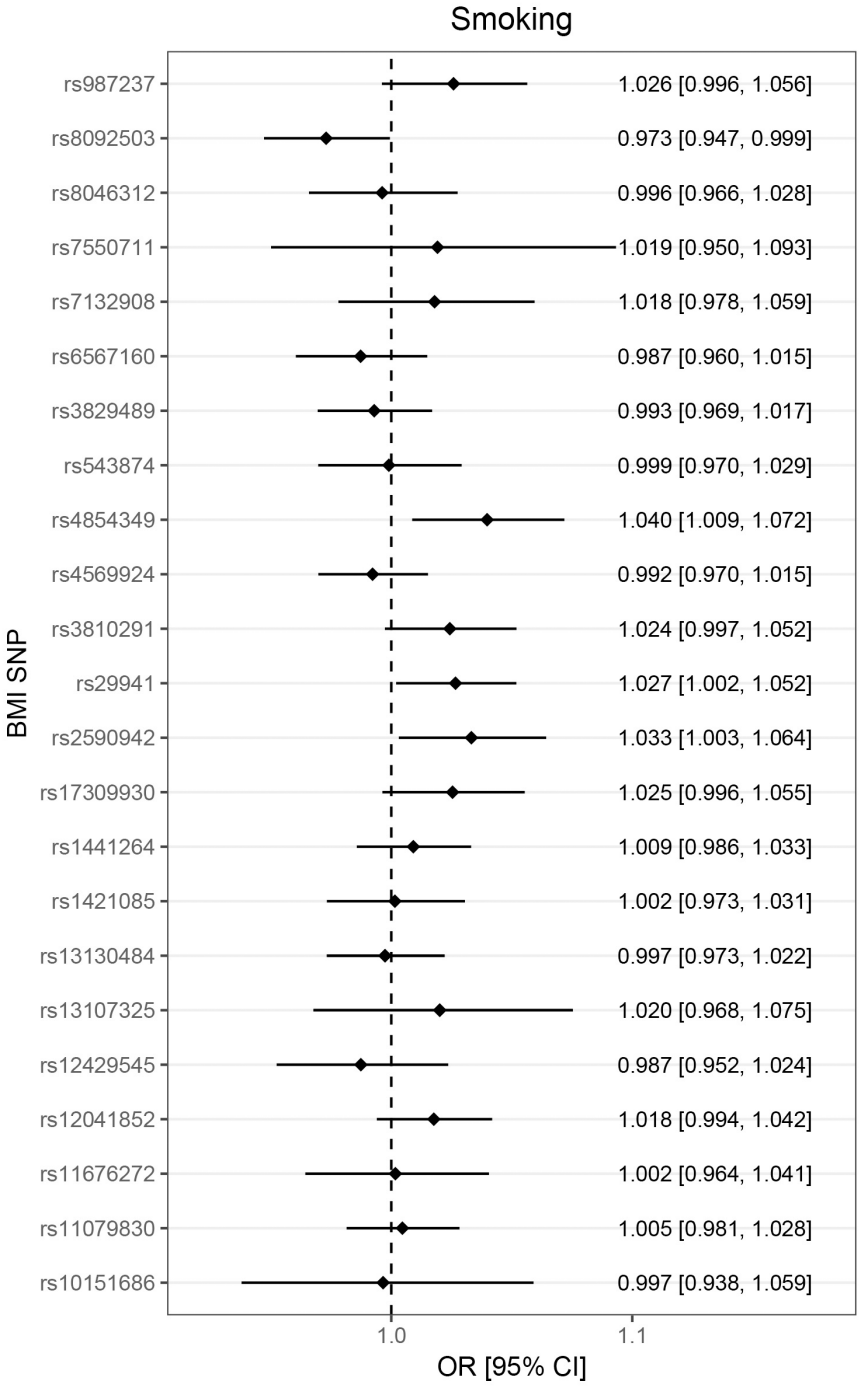
Association of childhood adiposity-related genetic variants with “ever smoker.” Lines represent 95% confidence intervals. Note that because of the large number of SNPs investigated, the threshold for nominal significance was set to *p* < 0.01. No SNP was alone associated with smoking, but the combined score showed a positive association. BMI, body mass index; CI, confidence interval; OR, odds ratio; SNP, single nucleotide polymorphism.

The heterogeneity test did not support heterogeneity in the data (*p* = 0.053 and 0.342, for the set of 23 SNPs and the set of 13 SNPs, respectively). However, scatter plots indicated that rs3810291 (nearest gene *ZC3H4*) might add heterogeneity (Fig 1, upper right panel). We checked phenotype associations of this SNP in PhenoScanner [49] but did not identify any reported significant (*p* < 0.01) effects on traits not related to adiposity and therefore kept this SNP in the analysis. The funnel plot shown in Fig 1 (lower right panel) implied directional pleiotropy biasing the results towards the null for the larger set of 23 SNPs, as confirmed by a nominally significant MR Egger regression intercept test (*p* = 0.036). For the smaller set of 13 SNPs, asymmetry was more difficult to judge but appeared less pronounced, as mirrored in an MR Egger regression intercept test (*p* = 0.615). We found that the larger set of 23 SNPs was associated with birth weight (0.07 SD increase in birth weight, 95% CI 0.04–0.10; per SD increase in childhood BMI) and with increased risk of smoking with an OR of 1.14 (95% CI 1.02–1.28) (Figs 2 and 3). The smaller set of 13 SNPs was associated with birth weight only (0.06 SD increase, 95% CI 0.02–0.10).

#### Robust analysis methods

The likelihood-based method provided similar results as the main analysis with an OR of 1.33 (95% CI 1.07–1.66) for the larger SNP set of 23 SNPs and an OR of 1.55 (95% CI 1.21–2.00) for the smaller set of 13 SNPs, indicating that the genetic associations with the risk factor were precisely estimated. Identical results to the main analysis were achieved when SEs were calculated using the random-effects model, indicating that the variability of estimates was not less than would be expected by chance [29]. For the larger SNP set of 23 SNPs, MR Egger regression yielded an OR of 2.76 (95% CI 1.40–5.44 and 1.24–6.11, using fixed and random weights for calculating SEs, respectively). For the smaller SNP set of 13 SNPs, MR Egger regression yielded an OR of 2.06 (95% CI 0.68–6.23 and 0.61–6.95, using fixed and random weights for calculating SEs, respectively).

**Fig 4:**
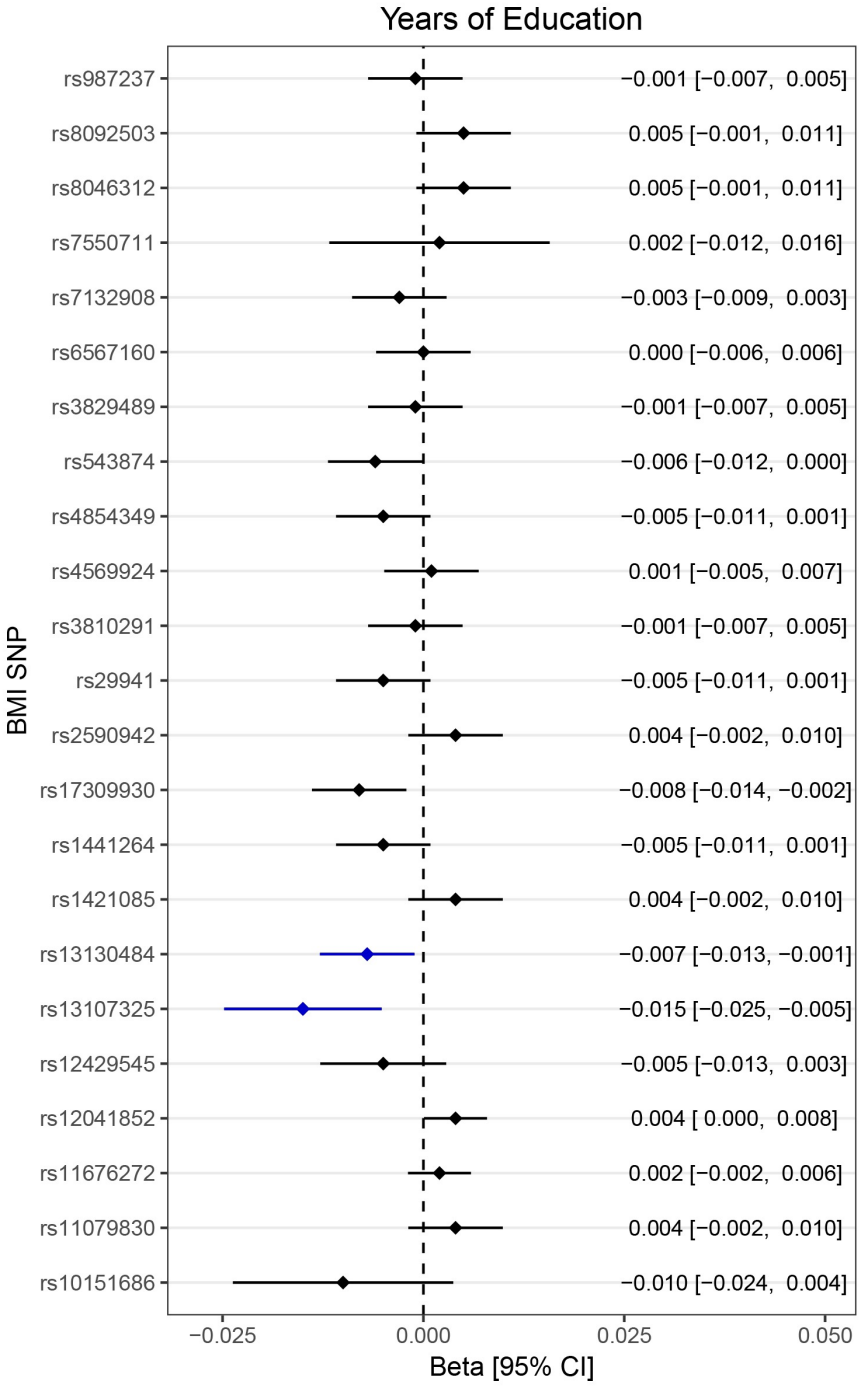
Association of childhood adiposity-related genetic variants with years of education. Lines represent 95% confidence intervals. Note that because of the large number of SNPs investigated, the threshold for nominal significance was set to *p* < 0.01. rs13130484 and rs13107325 were nominally associated with education and marked with blue color. BMI, body mass index; CI, confidence interval; SNP, single nucleotide polymorphism.

The simple median-based method and the weighted median estimate, only including the median 50% of the genetic instruments, resulted in similar estimates as the main analysis. Removing the 2 SNPs with nominal association to education (rs13130484 and rs13107325, Fig 4) slightly attenuated the IVW estimates to an OR of 1.26 (1.00–1.59) and an OR of 1.45 (1.11–1.90), respectively, for the larger and the smaller set of SNPs.

#### Multivariable Mendelian randomization

In regression models of the association of the 23 adiposity SNPs with T1D adjusted for genetic effects on birth weight, smoking, and education, we estimated that adiposity increases T1D risk with an OR of 1.65 (95% CI, 1.08–2.53) per SDS-BMI increase.

**Fig 5:**
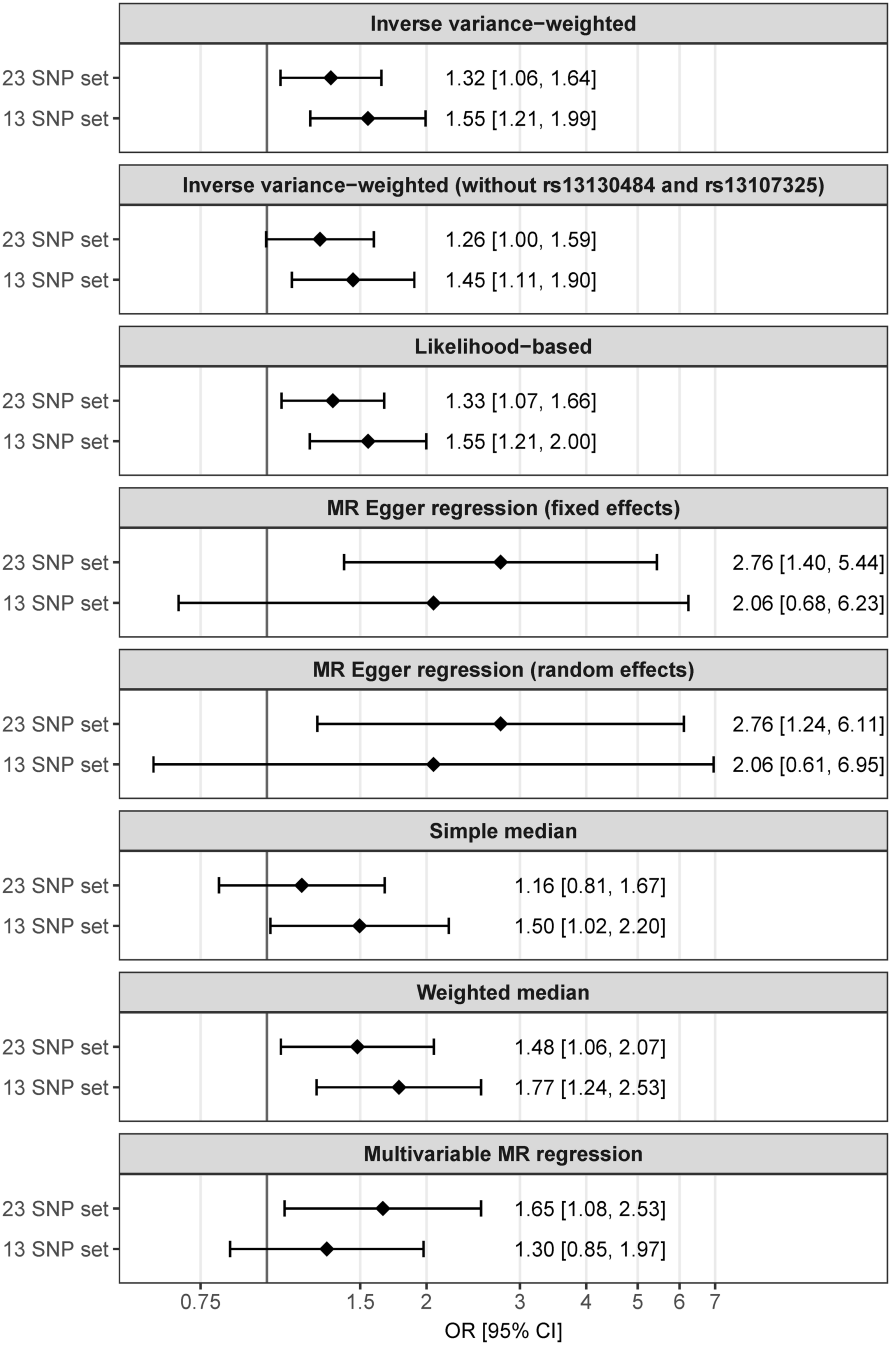
Summary of estimates from the different types of analysis. Lines represent 95% CIs. CI, confidence interval; MR, Mendelian randomization; OR, odds ratio; SNP, single nucleotide polymorphism.

Fig 5 summarizes results from the main and the sensitivity analyses.

## Discussion

The main finding of our study is that genetic variants predisposing to childhood adiposity may confer an increased risk of T1D, and we found evidence of a positive correlation between the effect on adiposity and T1D risk. Conditional on MR assumptions being satisfied, our study provides genetic support for a role of childhood adiposity in childhood T1D etiology. Our results support previous findings from observational studies and may indicate a link between the increase in global childhood obesity and T1D incidence in recent decades. We performed rigorous testing of MR assumptions and found evidence of (1) directional pleiotropy (biasing results towards the null) and (2) an association of the genetic score with smoking and birth weight. We therefore performed a series of methods robust to directional pleiotropy that confirmed the main results in favor of an effect of childhood adiposity on T1D independent of measured and unmeasured confounding effects.

### Biological mechanisms

Previous studies investigating a relationship between childhood adiposity and T1D using observational techniques in case-control and cohort studies have been contradictory [19–22,24,50,51]. However, in the meta-analysis by Verbeeten et al. [23], the pooled OR estimate for T1D risk per SD increase in BMI was 1.25 (95% CI 1.04–1.51), which is overlapping to our estimates from IVW and MR Egger regression. Different theories as to how childhood adiposity could contribute to the pathogenesis of T1D have been suggested. Obese individuals have a higher expression of proinflammatory adipokines, such as interleukin-6 [52] and tumor necrosis factor alpha [53]. These have been suggested to contribute to chronic subclinical inflammation promoting autoimmunity [54]. Leptin, an adipokine that is increased in obese persons [55], has been shown to accelerate immune-modulated destruction of beta cells in mice [56], and obese individuals have lower concentrations of the adipokine adiponectin that was shown to protect beta cells from apoptosis [57] and autoimmunity in animals [58]. Together, these effects could contribute to autoimmunity and beta cell death [54,56,57]. In contrast, the “accelerator hypothesis” [12] and the “overload hypothesis” [13] suggest that obesity contributes to insulin resistance and increased insulin demand. This would lead to beta cell stress and apoptosis, which could induce autoimmunity and cause T1D to present at an earlier age. Even if adiposity by itself does not cause T1D but rather accelerates disease onset, any delay in disease progress achieved by reducing adiposity would greatly benefit children at risk of T1D, as the likelihood of complication increases over time [59].

### Potential bias from pleiotropic effects

There are few established early-life risk factors for T1D. We chose to include 3 potential confounders in the present study where genetic data are available: birth weight, education level, and smoking. Genetic variants linked to increased birth weight are reported to be associated with higher risk of childhood obesity but lower risk of cardiometabolic disease in adulthood, including measures of insulin and glycemic traits [32]. The association of birth weight with T1D is unclear [60]. Further, some studies have pointed at an increased risk for T1D with higher socioeconomic class in Westernized countries [61]. A meta-analysis by Behl et al. [62] did not provide evidence for an association of parental smoking habits with T1D risk in their offspring. The average age of diagnosis in the present study was less than 8 years, and length of education and personal smoking habits may be irrelevant at this young age. However, parents share half of their genomes with their children, and we therefore explored these potential confounders. We removed 3 SNPs that were each associated with 1 of these confounders from the analysis. We also identified an overall association of the genetic instrument with increased birth weight and increased odds of smoking and therefore conducted an analysis adjusted for these variables, which supported our main results.

Our study includes a number of statistical analyses to detect bias from pleiotropy (i.e., effects of the genetic instrument not mediated by adiposity), and we detected that our main analysis may have been biased toward null results. In directional pleiotropy-robust MR Egger regression, we confirmed our main results.

These analyses did not reveal evidence of pleiotropic effects in any specific SNP, but we want to highlight a few interesting loci, although their associated genes have not been conclusively identified. The closest gene to rs13130484 with strong association with both childhood adiposity and T1D, *GNPDA2*, is involved in the hexosamine biosynthesis pathway, which is involved in cellular metabolic sensing and insulin resistance [63]. *TMEM18* is the closest gene to the SNP rs4854349, which showed the largest effect size for both SDS-BMI and T1D. Although variants related to this gene have been associated with BMI and insulin concentrations, little is known of its function. A study in *Drosophila melanogaster* implicated *TMEM18* in insulin/glucagon regulation [64]. The variant rs11676272 is among the SNPs most strongly associated with childhood adiposity and is also nominally associated with T1D. It is located in the gene *ADCY3*, which has roles in intracellular signaling, including the insulin secretion pathway [65]. Although *ADCY3* has been associated with locomotor activity, food intake, and leptin sensitivity in mice [66], the exact mechanisms for its effect on obesity are unclear.

Despite the possible involvement of the *GNDPA2*, *TMEM18*, and *ADCY3* loci in insulin resistance, insulin/glucagon regulation, and insulin secretion, respectively, we chose to retain them in the IV analysis. The prevailing paradigm puts autoimmunity at the core of the development of T1D, and impaired insulin secretion may likely occur secondary to the autoimmunity [5]. As insulin is a key regulator of lipogenesis, it is not surprising that genes involved in insulin secretion and signaling are amongst the important candidate genes for the obesity phenotype. None of the SNPs were identified as outliers in heterogeneity tests, although low power limits their interpretation.

### Strengths and limitations

Strengths of our study include the IV analysis design, the large study sample, and the detailed investigation of possible violations of MR assumptions. Our study selected genetic instruments associated with childhood rather than adult adiposity to test for effects specific to early-life BMI in patients diagnosed with T1D before 17 years of age. The limited age range in the T1D analysis limits heterogeneity in the diabetes phenotype and also restricts the exposure period to childhood.

Limitations of the present study include those that apply to all MR studies; the IV assumptions are stringent, yet it is often impossible to ascertain that genetic instruments fulfill the criteria. For many of the instruments used in the present study, the underlying gene is unknown. Without knowledge of the biological mechanisms, it is difficult to ascertain bias from pleiotropy. Yet, if rigorous sensitivity analyses support the main findings (as in our study), a causal effect is plausible [29]. Some of the sensitivity methods add additional assumptions, such as that potential pleiotropic effects are not related to instrument strength (InSIDE) [43]. In general, all point estimates from the sensitivity analysis yielded consistent or more extreme estimates, but in some instances with larger CIs overlapping the null.

Whilst the T1D dataset we used was the largest publicly available GWAS to our knowledge, MR analyses require large samples to yield precise results, and our study would have benefited from increased sample size. Finally, our results need to be confirmed, preferably in a large, independent study to exclude a chance finding.

### Conclusion

We have conducted an MR study investigating the relationship between childhood adiposity and T1D. Our results are in line with a role for adiposity in T1D etiology and show consistency in sensitivity analyses. As MR assumptions are not fully testable, our results, although confirmed in sensitivity tests, provide moderate but not conclusive evidence of causation. Given the worldwide increasing rates of childhood obesity and T1D, concerted global efforts to reduce excess body weight in early life to prevent adverse health consequences are strongly supported by our findings.

## Abbreviations

1958BC: 1958 Birth Cohort
BMI: body mass index
CI: confidence interval
EGG: Early Growth Genetics
GIANT: Genetic Investigation of Anthropometric Traits
GRID: Genetic Resource for Investigating Diabetes
GWAS: genome-wide association study
InSIDE: Instrument Strength Independent of Direct Effect
IV: instrumental variable
IVW: inverse-variance weighted
MR: Mendelian randomization
OR: odds ratio
SD: standard deviation
SDS-BMI: age- and sex specific standard deviation score of childhood body mass index
SE: standard error
SNP: single nucleotide polymorphism
SSGAC: Social Science Genetic Association Consortium
T1D: type 1 diabetes
TAG: Tobacco and Genetics
UKBS: UK Blood Services
WTCCC: Wellcome Trust Case Control Consortium
